# Chronic low‐grade Inflammation in Obesity as a Potential Biomarker for Alzheimer's Disease

**DOI:** 10.1002/alz70855_106637

**Published:** 2025-12-24

**Authors:** Amanda Rodin, Deepesh Khanna

**Affiliations:** ^1^ KPCOM, Nova Southeastern University, Clearwater, FL, USA

## Abstract

**Background:**

Obesity is a multifaceted disease that dramatically impacts an individual's quality of life and, when prolonged, can lead to an increase in several inflammatory markers, leading to chronic low‐grade inflammation. This review seeks to compile scientific literature related to obesity and its associated inflammatory markers that may be related to the onset of Alzheimer's Disease (AD).

**Methods:**

Literature was acquired using PubMed, Google Scholar, and NIH distributed from 2020‐2024. Obesity, Alzheimer's Disease, inflammatory markers, neuroinflammation, insulin resistance, and immune signaling pathways were keywords used. Inclusion criteria involved relevant peer‐reviewed studies published in English, within the last five years that also included populations characterized as obese. Exclusion criteria comprised pertinent studies that were published outside the time frame, in addition to studies that were not peer‐reviewed journals or contained non‐relevant populations.

**Results:**

Studies suggest that low‐grade systemic and local inflammation observed in obese individuals is associated with the development of other chronic conditions, as well as an earlier onset of AD. This can be attributed to the release of pro‐inflammatory markers and hormones caused by the accumulation of adipose tissue, while M1 macrophages further induce the inflammatory state by releasing more cytokines and activating microglia. Reactive oxygen species disrupt the blood‐brain barrier, leading to ischemia and apoptosis of neuronal cells, contributing to the AD phenotype. Additional studies have shown that insulin resistance, associated with obesity, leads to amyloid deposits and plaque buildup in the brain, which impacts brain cell function, a biomarker for AD.

**Conclusion:**

While inflammation is believed to be the primary cause of AD, to gain a deeper understanding of the relationship between obesity as an increased risk factor for developing AD, further research must be done. Obesity as a modifiable risk factor for the development of other chronic conditions emphasizes the significance of research in this area and the need for urgent intervention, especially among certain ethnic and racial groups, where African American women are the most likely to be affected by both obesity and AD.

